# Man’s Place in Creation

**Published:** 1875-07

**Authors:** P. H. Hayes


### MAN'S PLACE IN CREATION.

P. H. HAYES, M. D.

The Greeks, the most learned nation of antiquity, could not account for themselves. Some of the states declared their first fathers grew from dragon's teeth sown in the soil; others having no dragon's teeth to fall back upon, said their's sprang directly from the bosom of their mother earth.—After all, these ridiculous fancies of the older Greeks suggest the wonderful adaptation of our animal body to the earth we inhabit. I once attempted to wade a deep stream, and when up to my neck I experienced the strange feeling of having no weight; and though I could still touch my feet to the earth I could not move—there was no weight, therefore no resistence, and the current carried me helplessly off. The diameter of the Asteriod Pallas is but 80 miles, our earth 8000.—We stand by our weight, i. e., the attraction of the earth for our bodies, and this attraction is directly as the quantity of matter, and to this we owe our power to stand, to walk, to plough, to reap, to gather into barns and whatever else of bodily activity is necessary to life and happiness. If we were translated to Pallas, a mere foot-ball of a planet, we should be so light that we would be like a person wading in deep water. On the other hand, Jupiter contains more than 300,000 times the quantity of matter of our globe, and were we translated thither, our bodily frame would be so heavy that our weight would be like lead, and neither our bones or muscles would have strength in proportion to our weight. We might even be crushed by the simple power of attraction. So much is man a part of the earth he inhabits, that not only the very materials of his body are drawn from its bosom, but these in just such quantity as to give to his body the exact weight and strength of material in every part.—The very atmosphere is in its pressure exactly adapted to the tissue of the lungs and to the strength of the blood vessels and to their fluid contents. So, also, the waves of vibration caused by sounding bodies, find in the apparatus of hearing the exact organic structure to reciprocate these vibrations.

All that has been said as to the adaptation of our bodies to our planet, applies with equal truth and force to animals. Alike do man and animals breath the same air, have essentially the same organs of hearing, of sight, so exquisitely adapted to the light, of digestion, adapted to what the earth grows for food, of blood vessels, heart and circulation. Nay, so close are the analogies between the higher animals and man, that it is said if the brain of a Chimpanzee baby and that of a human baby were placed side by side before you on the table, no difference would be obvious to you. So the bible tells us man and brute alike came from the dust of the earth. "And the Lord God formed man of the dust of the ground." "And out of the ground the Lord God formed every beast of the field and every fowl of the air." God does the same thing now—creates and re-creates every human being and every creature, day by day from the dust of the ground; for, fed by the earth is every plant and every grain and every beautiful fruit upon which we live.

But when we have proved our utmost as to the common origin, and the common adaptations and common and corresponding members and offices in man and animals, when we have done our utmost to prove man an *animal*, we have done nothing to prove that he is nothing more. Nay, the stronger the proof that he is like the animals below him in everything belonging to *organization*, the more striking is the proof that as to the constitution of his soul he is another being entirely. So the sublime record again declares: "And God said let us make man in our image, after our likeness." If then the breath, the spirit of very God enters into the constitution of our souls and makes the distance between us and brutes in this respect so vast and sublime, let us try to sum up for ourselves some of the grand and more obvious elements of our superiority in soul to those animals we are so very like in physical constitution.

We have not far to look: And God said let them have *dominion* over the fish of the sea, the fowl of the air, and over every living thing and over all the earth.

How marvellously has mind conquered nature! The sterile desert has by the patient hand of man been made literally to bud and blossom as the rose, and to yield the grain and fruit for man's sustenance. Salt Lake city, now a blooming garden, is a redeemed alkali desert. So complete is the triumph of machinery as applied to agriculture, that a husbandman plants and sows in a sort of gig, and harvests his grain in a sort of chariot, yet within the memory of old men, the farmer bent his back all day and cut his grain by handfuls.

Man's dominion over every creeping thing is instanced in the silkworm, which year by year is made to produce the most costly and beautiful fabric by which woman drapes and adorns her person.

Even the insects are subdued. The Spanish fly is made to do duty in medicine, and the cochineal insect furnishes one of the most brilliant dyes. But nothing here compares with man's dominion over the honey-bee, which under the management of experts is made to build all its combs for raising brood and storing honey in regular lines and exact sizes and within frames, and so perfect has our knowledge become of the instincts and economy of these creatures that they can be perfectly controlled—made to raise their brood in one place and put their clean, new, white honey in another; they can be conquered and handled like flies, all their combs taken out and examined, and a single hive be made to yield five hundred pounds in a season in place of thirty or forty pounds fifty years ago, and, finally, instead of murdering these creatures over the sulpher pit to get their stores, we take only the honey they have to spare in neat little boxes, from which every single bee is not only permitted to escape, but is politely urged to do so.

Man's sovereignty over the higher animals of a hundred times his strength, is something grand. A friend says: "I was once riding in Syria with a few companions, when suddenly as we came around some hills upon a vast plain, there we saw a large herd of camels feeding, and under the charge of a single boy who could not have been more than seven years old. The boy was frightened and began to cry—for in that country an apparition of horsemen means plunder—but instantly recovering himself he mounted the leader of the herd and with many a thwack with his club and loud calls, he soon had his whole herd of great beasts in motion and off and away over the plains. 'Who but Allah!' exclaimed an Arab in our party, 'makes a little Adam like that do what he pleases with those big beasts?'" Any day you may see a little Adam on horseback in your streets, or upon the farm, who instinctively feels and is proud of his dominion over the horse. The race of dogs has been so wonderfully improved and brought into the service of man that single specimens of the finest blood are bought and sold for a thousand dollars each.

Again, there is a true divinity in the manner in which man has asked and answered the question how? It has brought him from a wigwam to a parlor—given him for the stylus the printing press, it has bridged the distance from a stone hatchet to a steam engine—it has given us a substantial cable by which we may speak to our friends and partners in business, in London, Paris and Vienna, and be answered in less than an hour.

Take a single department of art—that of surgery, which so wonderfully, so ingeniously, so sublimely comes forward to the relief of human suffering. A few hundred years ago all known surgery was almost wholly practiced by barbers and confined mostly to bleeding, to the compounding of unguents for wounds, and to a few most painful and clumsy operations. At this very moment it is still true in some parts of the world. But enter now the office of any distinguished surgeon and you are amazed at the case after case of beautiful instruments in his armamentarium, and you are impelled to gaze, and still the wonder grows, if one small head can carry all the surgeon knows. And if you shudder at the pain these instruments must cause—wait a moment and our surgeon will tell you he can perform the most painful operation known in his art, and put his patient to sleep while the process is going on. Yet the question *how* to abate or destroy pain in surgical operations had for hundreds of years been asked and so thoroughly failed to be answered, that so late as 1844, the greatest living surgeon, Velpeau, of Paris, declared that to avoid pain in surgical operations, is a chimera that we can no longer pursue in our time. While the American edition of his book was yet damp from the press, Dr. Horace Wells, of Hartford, had discovered the anesthetic powers of nitrous oxide, and proved that "to avoid pain in surgical operations" is no chimera. Dr. Wells' great discovery was followed in 1846 by that of Drs. Morton and Jackson, of Boston, proving that sulphuric ether is also a perfect anesthetic; and, in 1847, Prof. Simpson, of Edinburgh, discovered that chloroform was a much more powerful anesthetic than either the nitrous oxide or the sulphuric ether.

Who shall now say what is impossible, and who so skeptical as not to see and to feel the divinity within him, and when shall we cease to praise Him, who permits us to enjoy such attributes of power and dominion in the use and enjoyment of all his works.

